# Constrained by Design: Influence of Genetic Encodings on Evolved Traits of Robots

**DOI:** 10.3389/frobt.2021.672379

**Published:** 2021-06-15

**Authors:** Karine Miras

**Affiliations:** Computer Science Department, Vrije Universiteit Amsterdam, Amsterdam, Netherlands

**Keywords:** locality, bias, phenotypic traits, behavioral traits, evolvable morphologies, evolvable robots, encoding

## Abstract

Genetic encodings and their particular properties are known to have a strong influence on the success of evolutionary systems. However, the literature has widely focused on studying the effects that encodings have on performance, i.e., fitness-oriented studies. Notably, this anchoring of the literature to performance is limiting, considering that performance provides bounded information about the behavior of a robot system. In this paper, we investigate how genetic encodings constrain the space of robot phenotypes and robot behavior. In summary, we demonstrate how two generative encodings of different nature lead to very different robots and discuss these differences. Our principal contributions are creating awareness about robot encoding biases, demonstrating how such biases affect evolved morphological, control, and behavioral traits, and finally scrutinizing the trade-offs among different biases.

## 1 Introduction

There are two main classes of genetic encodings, namely, direct encodings and indirect encodings; the latter are also known as generative encodings. Direct encodings represent each phenotype component independently in the genotype. Conversely, generative encodings allow the reuse of genotype portions that code for similar or identical phenotype components. When working with evolutionary algorithms, it is well known that an encoding benefits from a high locality (Gottlieb and Raidl, 1999; Rothlauf and Goldberg, 1999; Rothlauf and Goldberg, 2000). This means that small changes in the genotype should result in smooth changes to the phenotype, and thus smooth changes to the fitness (Jones and Forrest, 1995). The lack of such a property could cause good quality parents to produce very low-quality offspring, thus leading to local optima around these parents. In particular, generative encodings often present a low locality (Gottlieb and Raidl, 2000; Rothlauf and Oetzel, 2006; Rothlauf, 2006). This scenario is undesirable, once generative encodings afford very desirable properties, like reuse and regularity (Doursat et al., 2013). Reuse is of utmost importance because it allows simplifying the optimization problem by solving small parts and then reusing them together in different contexts. For instance, while only 30.000 genes code all traits of the human body (Deloukas et al., 1998), our brain by itself has trillions of neurons (Dellaert, 1995). Because of this reuse capacity, evolution can, for example, discover a limb only once and regularly repeat this limb multiple times in the body of a creature. The importance of reuse is corroborated by research on modularity and its relevance for evo-devo (Bolker, 2000; Kuratani, 2009).

For the evolution of robots, generative encodings are commonly employed, sometimes focusing on the controller (Harding and Miller, 2005), and sometimes evolving both morphology and controller (Jelisavcic et al., 2018). In his seminal work, Sims applied directed graphs to conjointly evolve morphology and controller of virtual creatures (Sims, 1994). Since then, many other examples have appeared in the literature investigating diverse topics, like body-brain co-evolution itself (Hornby and Pollack, 2001), soft-robots (Cheney et al., 2014), physical robots (Lee et al., 2013), reconfigurable organisms (Kriegman et al., 2020), environmental influences (Auerbach and Bongard, 2014), developmental neural controllers (Kodjabachian and Meyer, 1998), and even encodings that use low-level abstractions from biology (Bongard, 2002). Among all this literature, the two generative encodings most commonly used to evolve robots are CPPNs (Stanley, 2007; Stanley et al., 2009) and L-Systems (Lindenmayer, 1968).

With respect to encoding comparisons, studies in the literature have been excessively focused on performance (Komosiński and Rotaru-Varga, 2001; Yosinski et al., 2011; Collins et al., 2019), i.e., fitness-oriented studies. This is the case from simple encodings (Janikow and Michalewicz, 1991) for solving classical problems like the Knapsack (Colombo and Mumford, 2005), to neuroevolution (Gruau et al., 1996; Siddiqi and Lucas, 1998) and also complex applications like evolving robot morphologies coupled with controllers (Hornby et al., 2003). While scarce, a few studies extended their investigations to aspects beyond performance. For instance, two different studies (Veenstra et al., 2017; Veenstra et al., 2019) compared performance when using a direct and a generative encoding, but constraining body development to different limits of body parts, and showed that the generative encoding is more efficient in the initial stages of evolution. Furthermore, it has been demonstrated that though CPPNs are able to produce more regularity, i.e., repetitions in the phenotype, than a direct encoding, the bias of the CPPNs towards regularity can be a complication for applications that require some level of irregularity (Clune et al., 2011). Another interesting study (Tarapore and Mouret, 2015) discusses the trade-off between performance and phenotypic variation. Notably, this focus of the literature on performance is limiting, considering that performance provides bounded information about the behavior of a robot system.

It is relevant for our discussion to mention that an encoding and its operators influence which phenotypes have more probability of being sampled. This may impose a space topology where some parts are more easily accessible than others, which may naturally result in biases (Komosiński and Rotaru-Varga, 2001). Importantly, such biases can be associated with the low locality previously discussed and can have a powerful effect in constraining searches in different ways. Considering that two given encodings may produce different search spaces, they may present different phenotypic constraints, despite acting upon the same design space. This means that the choice of a particular encoding may be a limiting factor for a given application that does not suit the imposed constraints. For example, the effect of environmental influences on robot traits was investigated (Miras and Eiben, 2019), demonstrating the difficulty of inducing phenotypic differentiation through environmental changes. In fact, despite some environmental changes having caused clear degradation in evolvability, in some cases robots still converged to the same phenotype. Although this study was not conclusive about why this happened, another study hypothesized that this could be due to limitations of the encoding (Miras et al., 2020). Finally, and on the other hand, the existence of a constrained space could, for a particular application, be actually desirable.

In this paper, we investigate the effects of generative encodings on evolved robots beyond performance, analyzing phenotypic and behavioral traits. Our main contributions are a) create awareness about robot encoding biases, b) demonstrate how such biases affect evolved morphological, control, and behavioral traits, c) scrutinize the trade-offs among different biases, and d) demonstrate a mechanism to evade undesired biases.

## 2 System Description

Our experiments were realized using a simulator called Gazebo, interfaced through a robot framework called Revolve (Hupkes et al., 2018). Here, we refer to both morphology (body) and controller (brain) as the phenotype of a robot.

### 2.1 Robot Morphology

**FIGURE 1:**
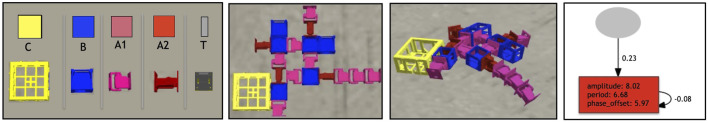
In the first box, the robot modules: Core-component, which carries the controller electronic board (C); Structural brick (B); Active hinges with servo motor joints in the vertical (A1) and horizontal (A2) axes; and Touch sensor (T). The polygons above the pictures of the modules are used to illustrate robot parts in the results section, and the letters are used to represent the modules in the robot encoding. Modules C and B have attachment slots on their four lateral faces, and A1 and A2 have slots on their two opposite lateral faces; T has a single slot, which can be attached to any slot of C or B. In the second box, an example of a robot **(top-down view)** before the simulation starts, while in the third, the same robot during a simulation. In the final box an example of a controller of a robot that has a single sensor and a single joint: the network controller has a single oscillator neuron (with a recurrent connection), and a single input.

Each morphology phenotype is composed of modules (Auerbach et al., 2014) as shown in Figure 1, and the shape of the morphology is determined by evolution. Each module has a cuboid shape and has slots where other modules can attach. The morphologies can only develop in two dimensions, that is, the modules do not allow attachment to the top or bottom slots, but only to the lateral ones. There are five different types of modules: core components, bricks, vertical joints, horizontal joints, and touch sensors. Any module can be attached to any module through its slots, except for the touch sensors, which cannot be attached to joints. Each module type is represented by a distinct symbol, and these symbols are used in the genotype encodings.

Previous work (Jelisavcic et al., 2017) demonstrated that this modular robot system functions in real hardware. Each module can be 3D printed, while the assembling of the modules and electronic parts (servos, sensors, board, etc.) is made manually. Production tutorials can be found in the link http://robogen.org/docs/video-tutorials.

### 2.2 Robot Controller

Each controller phenotype is a hybrid artificial neural network (Figure 1), which we call Recurrent Central Pattern Generator Perceptron (Miras et al., 2020). By hybrid we mean that we combine concepts from 1) CPGs, by having oscillator neurons; 2) Perceptrons, by having inputs connected to a single layer of neurons; 3) Recurrent neural networks, by allowing these neurons to have recurrent connections. In practice, the oscillator neurons generate a constant pattern of movement, and the sensor inputs can be used either to reduce or to reinforce movements, while the influence of these inputs can be remembered from each previous oscillation cycle. Every aspect of the network is defined by evolution, and the network is formed by two types of nodes: input nodes associated with the sensor modules; and oscillator neuron nodes associated with the joint modules. For every joint in the morphology, there exists a corresponding oscillator neuron in the network, whose activation function is defined by Eq. 1, which represents a sine wave defined by amplitude, period, and phase offset parameters. This activation function adjusts the output to fit the range of our servo motors, as proposed in (Hupkes et al., 2018).

$$O=0.5-\frac{a}{2}+\frac{\text{sin}\left(\left(2\text{*}\pi /p\right)\text{*}\left(t-p*o\right))\right)+1}{2}\text{*}a,$$ (1)

where, *t* is the time step, *a* is the amplitude, *p* is the period, and *o* is the phase offset. The parameters *a*, *p*, and *o* can vary from 0 to 10.

The different oscillator neurons can not be directly interconnected, and every oscillator neuron may or may not possess a direct recurrent connection. Additionally, for every sensor in the morphology, there exists a corresponding input in the network, and each input might connect to one or more oscillator neurons.

## 3 Methodology

### 3.1 Encodings

In independent experiments, we utilized two different types of generative encodings, namely: CPPN and L-System. Note that while the terms “encoding” or “representation” may or may not include both the genotype and its decoding (Eiben and Smith, 2003), also known as genotype-phenotype mapping, here we consider that it includes both. Because we, naturally, designed our own decodings for the CPPN and the L-System, it would be more precise referring to them as CPPN-based and L-System-based encodings. Nevertheless, for the sake of simplicity, we refer to them as simply CPPN and L-System.

#### 3.1.1 Compositional Pattern Producing Network

**FIGURE 2:**
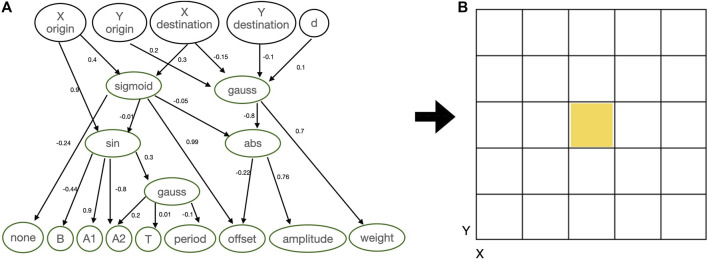
**(A)** Didactical example of CPPN: inputs are the coordinates of the substrate, while outputs are used to define modules to be placed in the substrate and also the parameters of the controller. **(B)** Morphology substrate.

In this case, the robot genotypes are represented using compositional pattern producing networks (CPPNs) (Stanley, 2007; Stanley et al., 2009), conjointly encoding both morphology and controller. A CPPN is a neural network that evolves to have multiple layers with diverse activation functions and is used to generate structures. A structure is generated by inputting the network with a context related to the structure, for instance, coordinates, and then using the outputs of the network to define the building blocks of this structure. Figure 2 shows an example of CPPN and the substrate that it intends to populate. The substrate is a 2D grid representing the robot morphology and has multiple cubes so that each cube may contain a module or not. The size of the substrate equals $z^{2}$, and here we define z=9, having the central cube always containing the head. Using the CPPN to define a morphology or controller trait is called querying, and it means inputting the normalized coordinates of a cube to the CPPN, so to use its outputs to define a trait. Additionally to the coordinates, the distance *d* between the center and the queried cube is also inputted. The possible outputs of the CPPN are the types of modules to be placed in a cube (or if the cube is supposed to stay empty), and parameters for the controller. In practice, the module selected by each query is the one with the highest value outputting from its respective neuron. As for the parameters of the controller, they are used exactly like they are outputted by the network.

The querying happens in two stages, and some inputs and outputs may be ignored depending on the stage. Stage one: 1) the querying starts from the head, as a turtle-reference is set to the central cube; 2) clockwise around the turtle-reference cube, every direction around it is recursively queried using *X* and *Y* destination; 3) the recursion stops when reaching the limits of the substrate, or when encountering a cube that is already occupied; 4) when a module is queried to be placed into an empty cube, if the new module is attachable to the module in the turtle-reference cube then they become attached, and the turtle-reference moves to the new module; 5) if the new module is a joint, the parameters for the controller are plugged to the controller accordingly, otherwise they are ignored. Stage two: 1) extra queries are made to define the weight between sensors and joints when these modules have already been queried, where *X* and *Y* origin represent a sensor and *X* and *Y* destination represent a joint; 2) to query the weight of a recurrent connection of a joint *X* and *Y* must be provided as the same for origin and destination; 3) the parameter weight must be above 0.05 for a connection to be created.

In the initialization, the CPPNs have no hidden neurons, and the inputs fully connect to the outputs using random weights drawn from a normal distribution with μ=0 and σ=1, while the activation functions are also randomly chosen. The activation functions utilized were sigmoid, sinusoid, Gaussian, tanh, absolute, and inverse. The reproduction is made by copying a parent sampled using a binary tournament and then mutating it with a probability of 80%. When selected to be mutated, a genotype can go through multiple different mutations, with probabilities: 50% for adding a new connection, 50% for deleting a connection, 20% for adding a new node, 20% for deleting a node, and 80% for mutating any weight, 50% for changing any activation function. More details about these operators can be found in (Stanley and Miikkulainen, 2002).

#### 3.1.2 L-System

In this case, the robot genotype is represented using an L-System (Lindenmayer, 1968), conjointly encoding both morphology and controller. L-Systems are parallel rewriting systems composed by a grammar defined as a tuple G=(V,w,P), where,

• *V*, the alphabet, is a set of symbols containing replaceable and non-replaceable symbols.
• *w*, the axiom, is a symbol from which the generative process starts.
• *P* is a set of production-rules for the replaceable symbols, having one production-rule paired with each replaceable symbol.

The particular encoding and decoding design for the current robot system were proposed and studied in (Miras et al., 2018b; Miras et al., 2020). In summary, each genotype is a distinct grammar instance, making use of the same alphabet. The alphabet is formed by symbols that represent types of morphological modules as well as commands for assembling modules and commands for defining the structure of the controller. In the decoding process, the grammar uses its production-rules to grow from its axiom into a more complex string of symbols, and finally, these symbols are translated into morphology and controller components. To initialize a genotype, for each production-rule, exactly one symbol is drawn uniformly random from each of five categories of symbols in the alphabet. This process is repeated *s* times, being *s* sampled from a uniform random distribution ranging from 1 to *e*. The crossover probability is 80%, and is done by uniformly random selecting full production-rules from the parents. The mutation probability of a genotype is 80%, so that one uniformly-randomly chosen production rule is mutated. For this production-rule, there is an equal chance of adding, deleting, or swapping one random symbol from a random position of it. More details about the operators can be found in (Miras et al., 2020), while the only parameter we used differently is the maximum number of groups of symbols e=3, and the maximum number of modules m=81. This was done to allow the L-System robots to grow as large as the CPPN robots can grow, given the substrate size of the CPPN.

### 3.2 Robot Traits

To access the phenotypic and behavioral traits of the populations we utilized a set of trait descriptors proposed by (Miras et al., 2018b; Miras et al., 2020), where more details about them can be found. The descriptor Stability of Speed was proposed in the current paper. Importantly, with the term “behavior”, we mean what results from the interaction between the phenotype (body and brain) and the environment.

#### 3.2.1 Behavioral Traits

1.Speed: Describes the speed (cm/s) of the robot along the *x* axis.
2.Balance: Describes the rotation of the robot in the *x*–*y* dimensions, so that perfect Balance belongs to both pitch and roll being equal to zero. The higher the Balance, the less rotated the center of the robot is. The center is defined as the center of mass of the head-link: the union of the head and a possible group of non-actuated modules connected directly to it.
3.Stability of Speed: Describes the stability of robot behavior in regard to speed. This value represents the difference between the speed of a robot on two occasions, and is defined by Eq. 2:

$$s=s_{d}-s_{s}$$ (2)

where $s_{s}$ is the speed of the robot using the standard evaluation time, and $s_{d}$ is the speed of the robot using double this time. “Standard” means this evaluation time was used during the evolutionary process. The closer this value is to zero, the more stable the behavior of speed is. Being stable means that the robot speed on one occasion is not different than the speed in another occasion, even if this second occasion is a longer period of time.

#### 3.2.2 Phenotypic Traits

1.Size: Total number of modules in the morphology.
2.Proportion: The 2D ratio of the morphology.
3.Limbs: The number of extremities of a morphology relative to its body size.
4.Length of Limbs: Describes how extensive the limbs of the body are.
5.Joints: Describes how movable the body is.
6.Symmetry: Describes the reflexive symmetry of the body around the head.
7.Coverage: Describes how full is the rectangular envelope around the body.
8.Branching: Describes how the attachments of the modules are grouped in the body, accounting for the ones that have module attachments to all of its slows.
9.Average Period: Describes the average of the parameter Period among the oscillators of the controller. The higher this value, the slower the oscillation pattern, and thus slower the movement of the motors.

### 3.3 Evolution

We use overlapping generations with a population size μ=100. In each generation, λ=50 offspring are produced. From the resulting set of *μ* parents plus *λ* offspring, 100 individuals are selected for the next generation using binary tournaments.

The evolutionary process is divided into three stages: 1) primary initialization: happens in generation 0, using the initialization operators of each encoding; 2) secondary initialization: happens from generation 1 to 49, optimizing for morphological novelty; 3) optimization: happens from generation 50 to 149, optimizing for speed. During the optimization, each robot was evaluated for 30 s. Finally, for each encoding, the experiment was repeated independently 20 times. A summary of the parameters for the evolutionary algorithm is provided here:

• Population size 100
• Offspring size 50
• Number of generations 150
• Experiment repetitions 20
• Evaluation time 30

The secondary initialization stage is inspired by (Buchanan Berumen et al., 2020), with the optimization searching for morphological novelty using Novelty Search (Lehman and Stanley, 2008). In this case, the fitness function is defined as N=n, where *n* is a measure of novelty which is calculated as the average distance to the *k*-nearest neighbors of an individual, for which k=10 and the distance is the Euclidean distance regarding the following morphological descriptors: Branching, Limbs, Length of Limbs, Coverage, Joints, Proportion, and Symmetry. The set of neighbors for the comparison is formed by the current population, plus an archive, to which every new individual has a 5% probability of being added, with the individuals added to the archive remaining in it until the end. This step of search space exploration is important because, in the forthcoming analysis, it will help to demonstrate that the observed biases are not simply fruit of an unlucky or biased initialization.

As for the optimization stage, the optimization concerns increasing speed in a task of directed locomotion on a plane terrain. The fitness function utilized is defined by Eq. 3:

$$f_{1}=\{\begin{array}{cc} s_{x} & \text{if}\;s_{x}>0,\\ \frac{s_{x}}{10} & \text{if}\;s_{x}<0,\\ -0.1 & \text{if}\;s_{x}=0, \end{array}$$ (3)

where $s_{x}$ is the speed of the robot. This function measures the speed of the robots (only) in the *x* axis. Additionally, it has penalties for robots that do not move, or that go to the wrong direction.

### 3.4 Abortion Mechanism

The abortion mechanism acts upon the reproduction operator of the encodings and is employed in only one of two types of experiments we carry out in this paper, aiming to evade the encoding biases. The mechanism is described hereby: 1) sample c=50 children of a parent; 2) measure the distance between each child and the parent using the Euclidean distance among the same descriptors utilized for the measure of novelty; 3) choose one child to be born and abort all the others; 4) the child chosen to be born is the child closest to the parent, as long as it is not identical—if every child is identical choose a random child. This means that a total of 250 children are sampled in the reproduction step of each generation, while only 50 are ultimately born to be evaluated.

Moreover, to simplify the comparison between parent and child, we eliminate the crossover operator (if existent), and therefore reproduction is performed by copying the parent and mutating it with probability of 100%.

### 3.5 Experimental Setup

**FIGURE 3:**
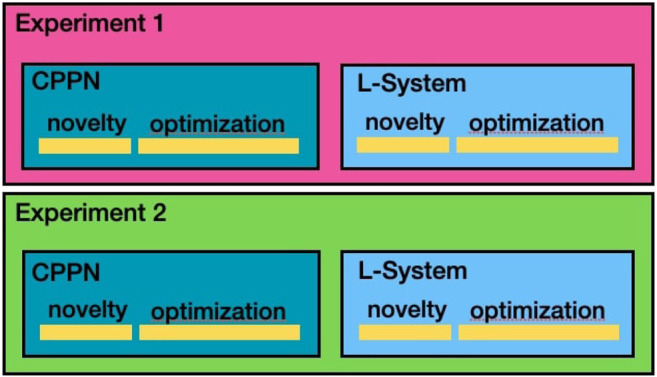
Illustration of the experimental setup.

We conducted two types of experiments using the same setup, except that in experiment 2 the abortion mechanism of the previous section is employed. Both experiment 1 and experiment 2 are divided into two sub-types, one using the CPPN encoding and another using the L-System encoding. Figure 3 illustrates the different experiments carried out. The code to reproduce all experiments is available on https://github.com/ci-group/revolve/releases/tag/Frontiers21constrained_1.1.

## 4 Results and Discussion

Hereby we analyze the influence of the two encodings on phenotypic and behavioral traits of the robot populations resultant from our experiments.

### 4.1 Novelty and Behavior

**FIGURE 4:**
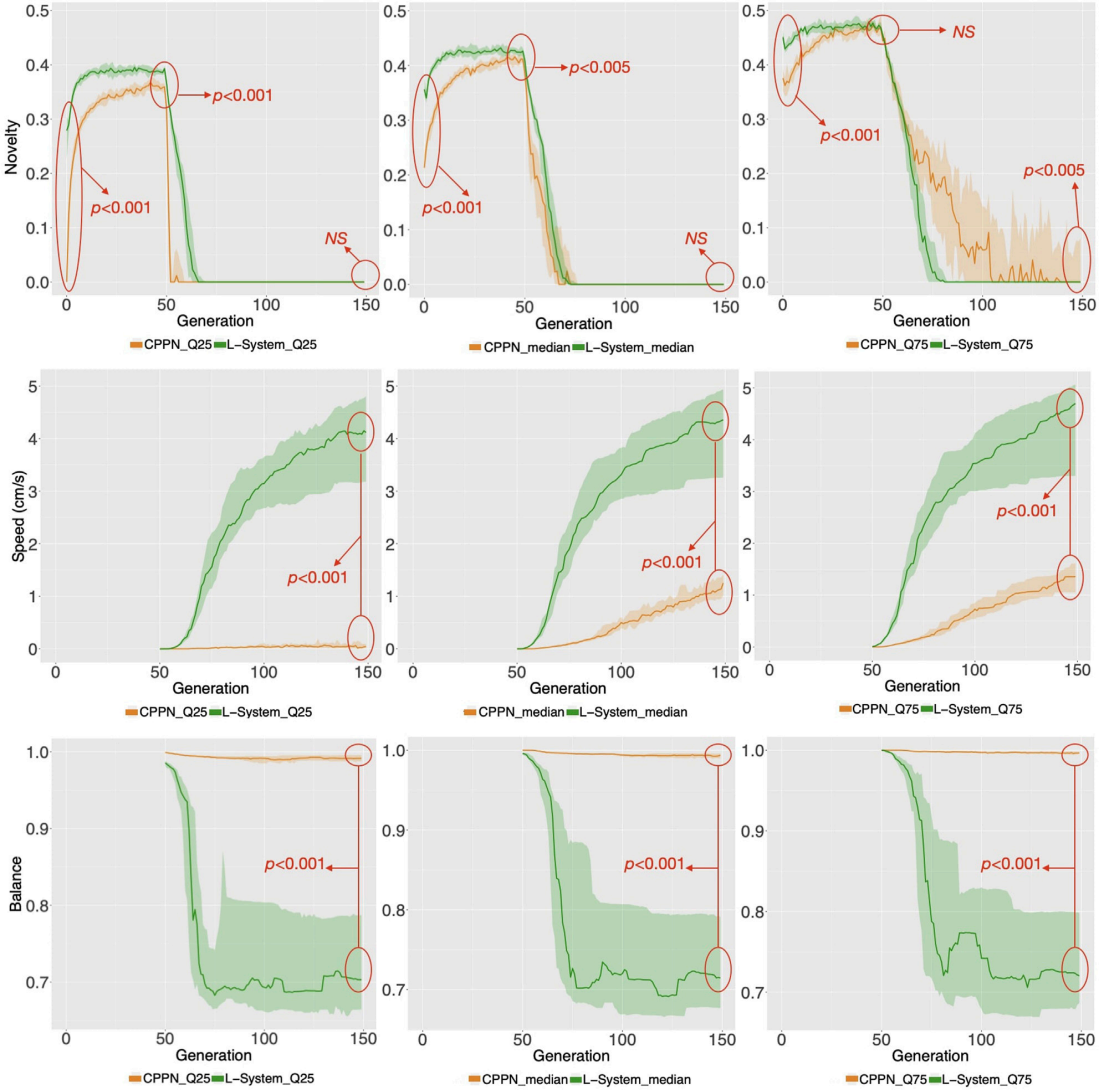
Distributions of novelty and behavioral traits for the populations. The aggregation of measures happens in two levels: Firstly, the plots on the left, middle, and right regard aggregations within the population of each run using the first quartile, median, and third quartile respectively. Secondly, the lines represent the median among the runs while the clouds around it represent their first and third quartile. The red arrows point to the *p* values of Wilcoxon tests comparing the two encodings in a given generation. NS means non-significant. Note that there is no behavioral data before generation 50, when robots were evolved for morphological novelty only.

**FIGURE 5:**
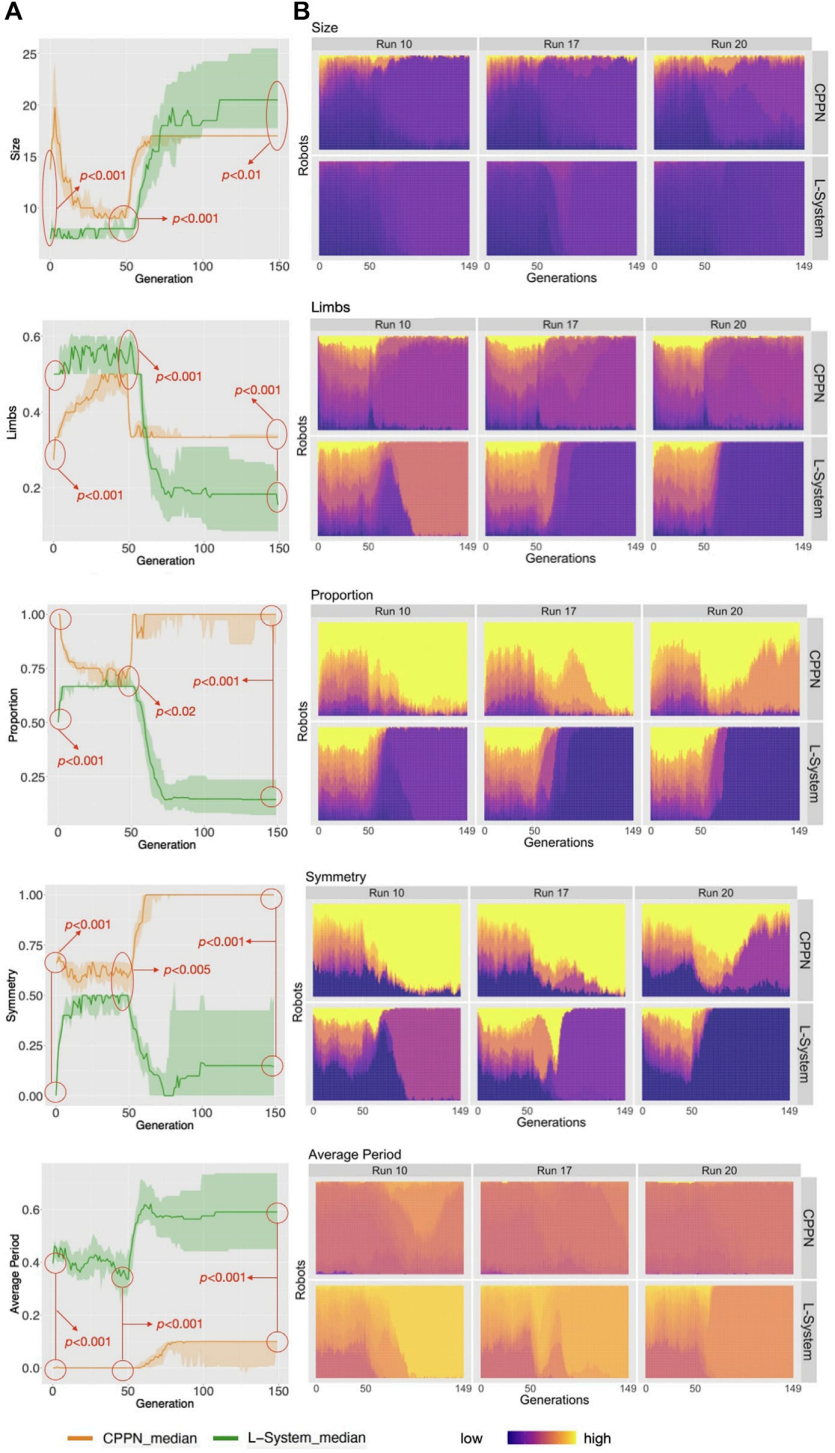
**(A)** Distribution of phenotypic traits for the populations. Aggregations and tests are the same as in Figure 4. **(B)** The heat-maps display the values for the robot traits of every robot in every generation, including three example runs.

**FIGURE 6:**
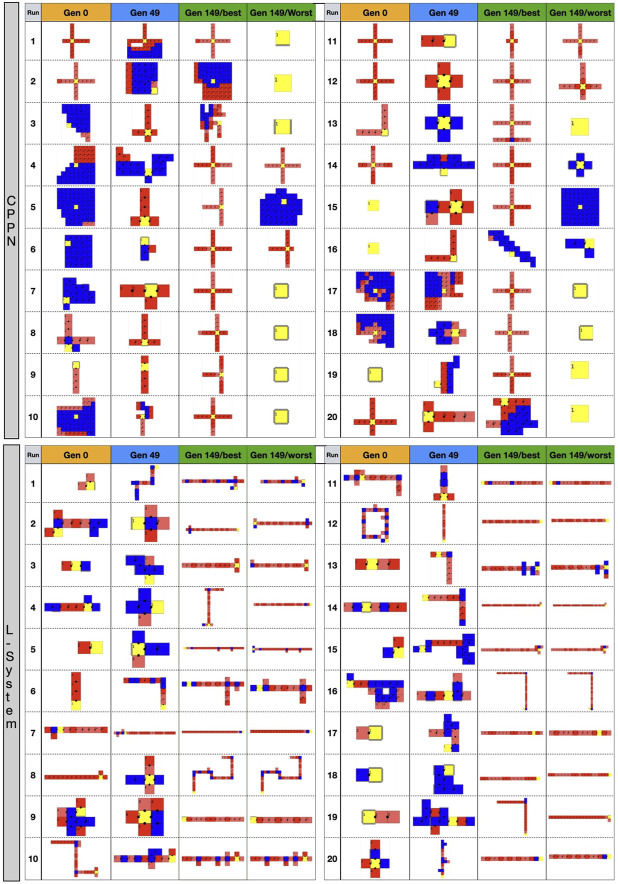
Top-down 2D illustration of robots in different stages of the evolutionary process. Gen 0: random robot from primary initialization. Gen 49: random robot from secondary initialization. Gen 149/best: best robot after optimization. Gen 149/worst: worst robot after optimization. Images size was adjusted to fit the cells.

We start by analyzing morphology holistically, comparing the multidimensional morphological novelty in the populations evolved using each encoding (Figure 4, top). This measure indicates how different from each other these individuals are within their populations. The same measure of novelty utilized to evolve the initial populations is applied here, but discarding the archive from the set of neighbors. The reason for not considering the archive is that we are now interested in the present diversity levels of the populations, and not in the history of the novelty search. The novelty in generation 0 is lower with the CPPN than with the L-System. This is true for the median as well as for the first and third quartile, and the difference is most intense for the first quartile. Moreover, even after evolving the populations towards morphological novelty, novelty is still lower with the CPPN. Though the two encodings naturally present an improvement in novelty in generation 49, this novelty is significantly lower with the CPPN for both median and first quartile. Finally, around 25 generations after switching the fitness from novelty to speed, which happened from generation 50 on, novelty dropped to zero in both the CPPN and the L-systems populations, and remained there for the median and first quartile. This drop seems to be caused by selection pressure for some given morphological traits. As for the third quartile, though novelty is a little higher with the CPPN, this novelty is deceiving and, as we are going to discuss soon, has a severe impact on the performance. The heat-maps of Figure 5 show that differently from the L-System, the CPPN presents in most generations some robots with extremely low or large size. Note that though these robots are different from the rest, they are not functional. In the column “Gen 149/worst” of Figure 6 we see that they often have no joints and are either blobs or only-head robots, which seems to be a consequence of a particular bias that will be discussed in the next section. As can be seen, the CPPN presents a higher morphological bias, i.e., often discovering very similar morphologies, that starts at the initialization and persists throughout the search to some extent. Nevertheless, these higher levels of morphological novelty of the L-System did not prevent its populations from converging morphologically almost as soon as the CPPN populations.

**FIGURE 7:**
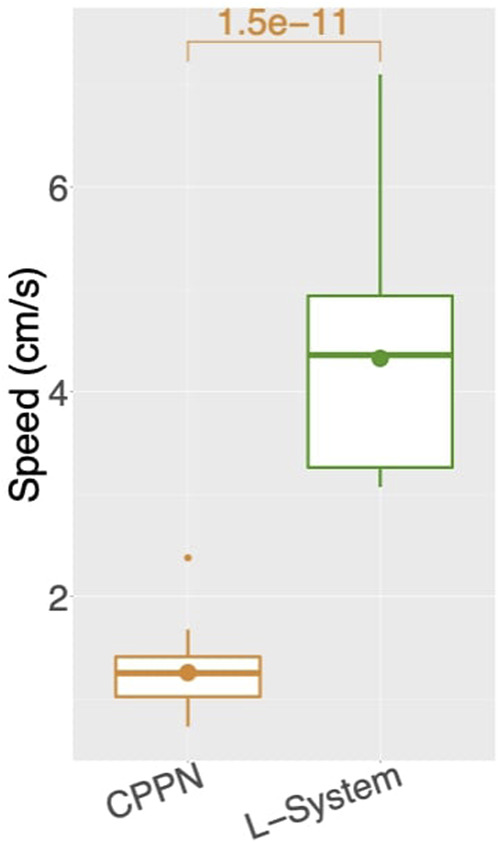
Average speed among runs: for each run, includes all robots of the final generation (149) aggregated using the median. Number above box is the *p* value of a Wilcoxon test.

Turning now to the performance on the task (speed), the two encodings exhibit a drastic difference (Figure 4, middle), with CPPN robots being on average more than three times slower for the median and third quartile. This difference is even more severe for the first quartile, where the CPPN has an average very close to zero. This first quartile difference seems to stem from dysfunctional robots discussed in the previous paragraph. Interestingly, both encodings present cases of “extreme runs,” meaning runs with an average very far from the averages of the other runs (Figure 7).

**FIGURE 8:**
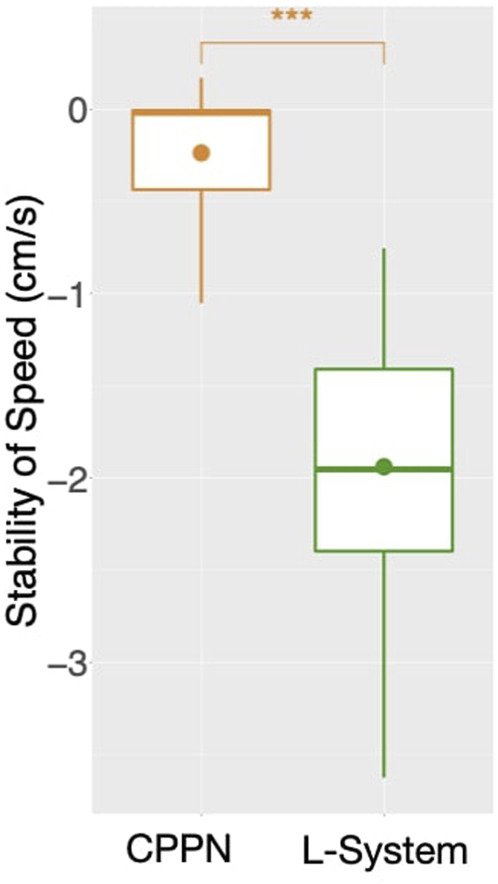
Average stability of speed among runs: for each run, includes all robots of the final generation (149) aggregated using the median. A marker above boxes represents a *p* < 0.001 for a Wilcoxon test.

While speed is a behavioral trait that we directly optimize through the fitness function, let us now observe a behavioral trait that emerged to accomplish the task, despite not having been directly optimized: Balance (Figure 4, bottom). This trait is interesting, because it can partially describe the gaits of the robots, and they happen to be very different when using each encoding. For median and both quartiles, whereas from the start CPPN presents and maintains almost maximum Balance, L-System quickly drops down to an average of around 0.7, and maintains that until the end. This distinction is corroborated when observing the robot gaits of the best individuals of the populations. A video with some of the best robots of the experiments can be seen and in Supplementary Material and here: https://www.youtube.com/watch?v=tZQ1dUoNHJY&feature=youtu.be. L-System robots most often locomote by rolling forward over their bodies, and only sometimes by rowing—simultaneously stroking with multiple limbs. In contrast, CPPN robots never roll, but most often row or walk. Note that rolling is a gait with low Balance, because by rolling the robot rotates its center. On the other hand, walking and rowing have a higher Balance, keeping the center relatively stable and producing a coordinated gait. This particular behavioral divergence is very relevant to our discussion because it helps us to elaborate on the trade-off between using each of the two studied encodings. Although the L-System robots perform better on the task, as measured by speed, their gaits are somewhat “reckless”. By reckless, we mean that their gaits are defined by messy irregular patterns of body motion. Though this recklessness may not necessarily be a problem, it is not hard to imagine a situation in which this would be concerning. For instance, the rolling robots have their heads often rotating and sometimes bashing them against the floor. Given that in our case the head carries the processing board, this rolling gait could increase the chance of damaging this board. Beyond this hypothesis, the irregularity in the gaits of the L-System snakes has a much more severe implication. During the optimization stage, the life-time of a robot is short, even though after deployment this lifetime would probably be much longer. Therefore, it is inherently desirable to have robots in the population that behave well during a short life-time, and that do not suffer significant degradation of their behavioral quality if their life-time is stretched longer. Nevertheless, when analyzing the stability of the behavior of speed with each encoding, we realize the L-System robots are very unstable (Figure 8). The measurements done with Eq. 2: show that while the CPPN robots have average stability close to zero, L-System robots have average stability close to −2. This means that while the CPPN robots present little difference in quality when living a longer lifetime, the L-System robots are in average 2 cm/s slower.

### 4.2 Phenotypic Traits

**FIGURE 9:**
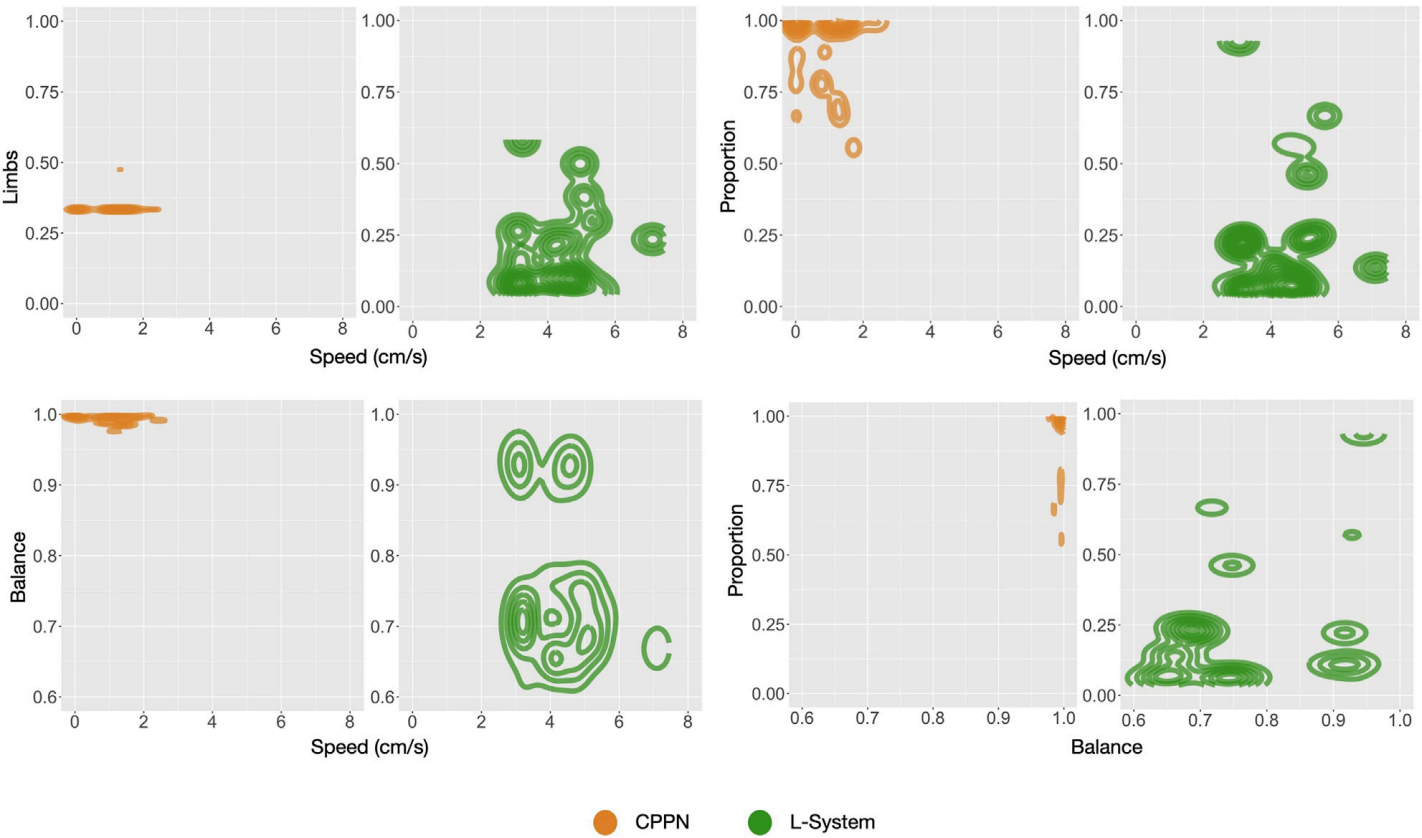
Density of robots for pairs of traits: includes all robots from each final generation (149) of each run all together.

As opposed to the previous section, here we analyze the independent phenotypic traits and their relations to behavioral traits. Figure 5 shows the distributions of four morphological traits and also one trait of the controller. Looking at these curves, what is most striking is that L-System robots are always different from CPPN robots, and this happens in all of the three stages of our evolutionary process: primary initialization, secondary initialization, and optimization. Additionally, in all cases except Size, traits that started at a higher value with one of the encodings remained higher until the end. For instance, Symmetry was lower with the L-System than with the CPPN at generation 0, generation 49, and generation 149. In the final populations, L-System robots were much larger, less proportional, less symmetrical, had fewer limbs, and a slower oscillation pattern. To illustrate better, Figure 9 depicts the relationship between phenotypic and behavioral traits. The density contours show that the L-System spreads not only to different areas, but to vaster areas of the space. Corroborating this observation, for all traits, the quartile deviations from the medians among the runs are wider for the L-System (Figures 4, 5). This means that with the CPPN, more often an evolutionary run converges to the same type of robot.

Considering the search chronologically, firstly we see changes in the average traits from generation 0 to generation 49—this is the case for traits that were included in the definition of novelty and used during the secondary initialization (Figure 5). These changes are reflections of the Novelty Search attempt to find diversity. Secondly, when optimization is taking place, we see all averages quickly dropping or increasing until stagnation. As expected, this agrees with the previously discussed averages of morphological novelty, in the sense that morphological convergence does happen. On the other hand, the curves of speed have not stagnated yet after the end of the evolutionary process. It is relevant to mention that, given that all phenotypic traits have converged but the speed continues to grow, we are led to conclude that what is still being optimized are traits that are not captured by our current trait measures. Moreover, because through visual inspection we see that most robots have converged to a particular body shape, as we will discuss soon, this speed growth can most likely be attributed to changes in combinations of control parameters, which happen to be less intelligible and thus harder to observe.

We shall now inspect closer the appearance of the robot morphologies. Figure 6 displays examples of robots in each of the evolutionary stages. We see that after the optimization stage, the L-System often produces I-shape robots, i.e., “snakes,” while CPPN often produces cross-shape robots, i.e., “spiders.” Moreover, the best robot and worst robot from a run frequently look the same or very similar with the L-System. By contrast, in most cases with the CPPN, the worst robot is a dysfunctional robot characterized by a blob or an only-head robot. This supports our previous observations about the CPPN maintaining a (deceiving) slightly higher diversity by sampling simple dysfunctional robots. Another aspect to note is that in the primary initialization the CPPN already frequently produces spiders and simple dysfunctional robots, though this happens less frequently after the secondary initialization. The same can be said about the L-System, but the simple dysfunctional robots are accompanied by snakes instead of spiders. Notably, while with the L-System it is unclear why simple dysfunctional robots are produced so often right from the initialization, in the case of the CPPN this phenomenon is more simply explainable. Because the CPPNs are randomly initialized—before any optimization takes place, it is very easy to obtain a neural network that (almost) always outputs (almost) the same results regardless of the inputs. In our particular case, outputting always the same results means the same module gets selected to be placed in every position of the substrate. Finally, given the nature of our decoding and how the modules are allowed to attach, when this happens, the most common morphologies inevitably become something close to or exactly like 1) spiders—when the joint neuron always has the highest value, 2) blobs - when the block neuron always has the highest value, 3) only-head—when the “no module” neuron always has the highest value, and 4) only-head with sensors—when the sensors neuron always has the highest value. See illustrations in Figure 6, run 11 generation 0 for spider, run 15 generation 149/worst for blob, run 16 generation 0 for only-head, and run 19 generation 0 for only-head with sensors.

### 4.3 Encoding Biases

**FIGURE 10:**
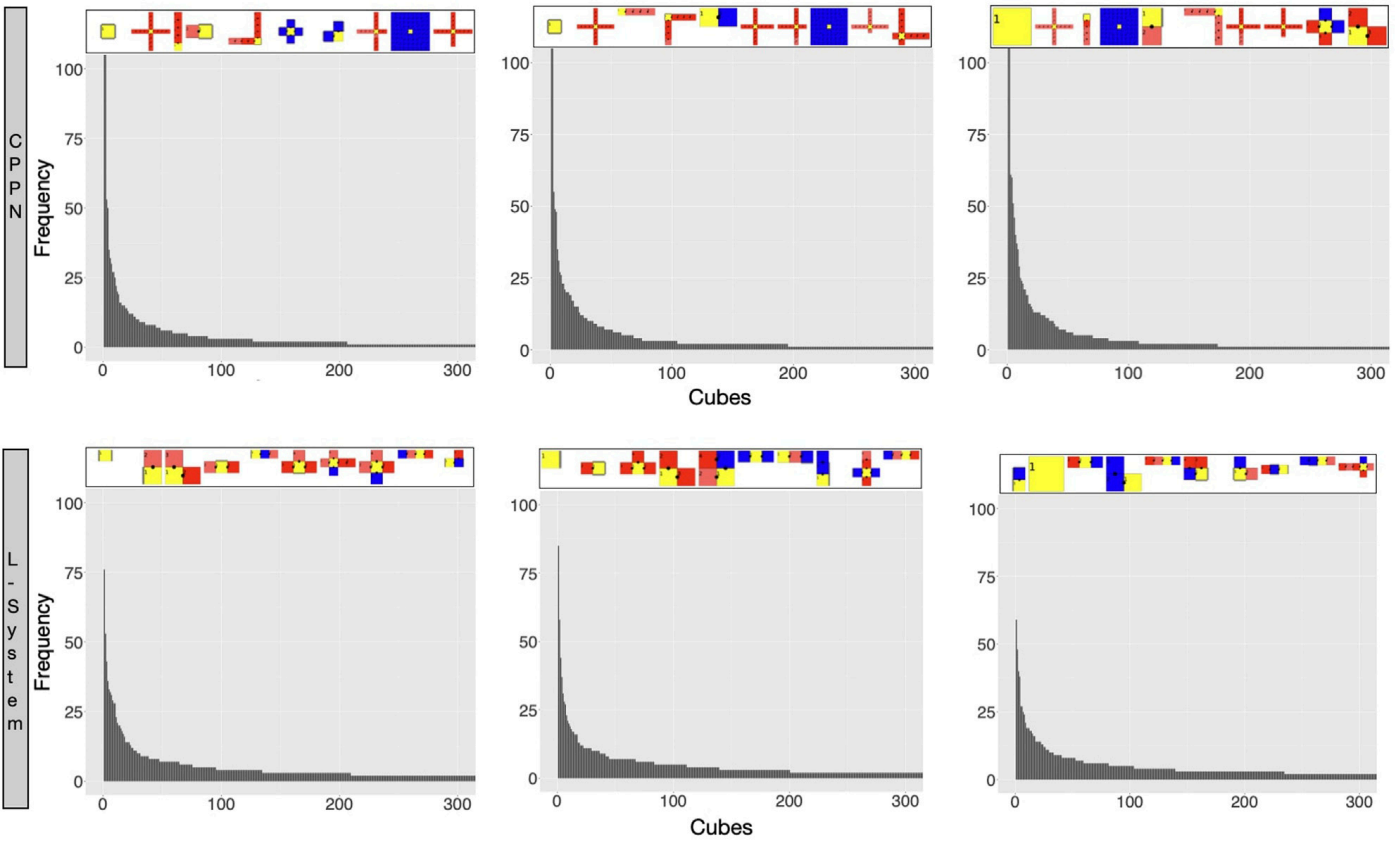
Robots most frequently sampled by the reproduction operators during the secondary initialization. Each bar in the histogram represents how many times robots that fit into that cube were ever sampled (born). Each cube is part of a large multidimensional cube where each dimension is one of the morphological traits divided into 100 bins. The robots on the top of the plot are the representative of the cubes with the highest frequency (ordered from higher to lower). Each histogram concerns one random run. Plots were scaled in *x* and *y* to 300 and 100 respectively.

So far we described the traits of robots produced by each encoding, and how they are biased. Let us now watch closely what these biases are, by inspecting the exploration of the search space (Miras et al., 2018b). Figure 10 displays the robots most commonly born during the secondary initialization stage of the evolutionary process when the population was being evolved towards novelty. This way, there was no selection pressure for convergence, but divergence. However, while a perfectly unbiased search would result in cubes being uniformly visited, what we see is an exceedingly skewed distribution, with some cubes having an extremely higher frequency than most others. In practice, this means that a small number of morphology shapes are born much more often than most other shapes. Note that though with both encodings we get skewed distributions, the very highest frequencies in the L-System cubes are lower than the ones within the CPPN cubes. Observing the robots at the top of the histograms we see that, with both encodings, these most commonly born shapes are very simple, have few modules, and are often dysfunctional. In the case of the CPPN though, one of these very often born robots has a shape that is not so simple neither dysfunctional, i.e., the spider.

At this stage, our observations begin to suggest that the predominance of the spiders after the optimization stage when using the CPPN is due to this bias. We could reason that if spiders continue to be so commonly born despite the efforts of Novelty Search, then it may also persist despite the efforts of a task optimization. Beyond that, because the other most frequently born shapes are simply dysfunctional, this grants the spiders even more advantage for the task of locomotion. As for the L-System, the most commonly born shapes are often dysfunctional as well, but the large snakes predominant after optimization are never part of these most commonly born shapes discussed in the analysis above. Nevertheless, the high frequency of the dysfunctional shapes may grant the snakes as much advantage as it does to the spiders. The difference is that the spiders have the extra advantage of being part of the most commonly born shapes themselves. The observation that large snakes are not part of the most commonly born shapes like spiders invites us to wonder whether the eventual dominance in the population of the large snakes is not simply a product of the bias, but of genuine selection pressure for rolling snakes in the current environment and task. Corroborating this thought, related work (Miras et al., 2018a) using the current L-System has shown that it is possible to obtain very diverse populations by combining the fitness of speed with rewards for diversity and the growth of limbs. They demonstrated that this way, though populations of robots become diverse to the point of resembling animal-like bodies and gaits, these robots were dramatically slower than the snakes. Moreover, let us remember that the spiders are also dramatically slower than the snakes. Another interesting aspect to be considered is that though a medium size snake (four joints) is present as the third most common robot with the CPPN, snakes never take over after the optimization. Perhaps what explains that is the fact that spiders are born even more often, or even it could be that four joints are not enough for an effective gate. Still, a more plausible explanation may be that the best shape is never defined simply by a bias, but by the interaction of the search space and the task.

**FIGURE 11:**
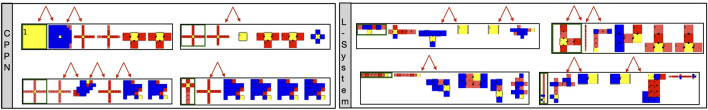
Examples of robot genealogy. From left to right, robots go from younger to older. The youngest robots were randomly sampled from generation 59 of a random run. The red arrows point out non-smooth morphological transitions from parent to child.

To conclude this section, we will investigate possible reasons for the observed biases. In Figure 11 we see examples of robots that were alive in generation 59. We chose a generation belonging to the optimization stage so that we could see examples of the “champion” robots, but we did not choose a too late generation so that we could still see their parents differing from them. What is prominent in these genealogy illustrations is how easy it is to transition from a simple dysfunctional shape into a more complex shape, and vice versa. In particular, it seems possible to transition from heads, blobs, etc. directly into spiders. For the sake of simplicity, in the case of the L-System that uses two parents, we depicted only one random parent. In any case, it is also possible that a small body shape has a child that is a large snake and vice versa. This effect of having non-smooth transitions between parent and child is called a low locality. This means that the heritability of both methods is not good enough, and then too often small changes in the genotype have enormous effects on the phenotype. We consider this to be a bias resulting from the interaction between the encodings with their reproduction operators, and advocate that awareness about this type of bias must be created.

### 4.4 Effects of Abortion

**FIGURE 12:**
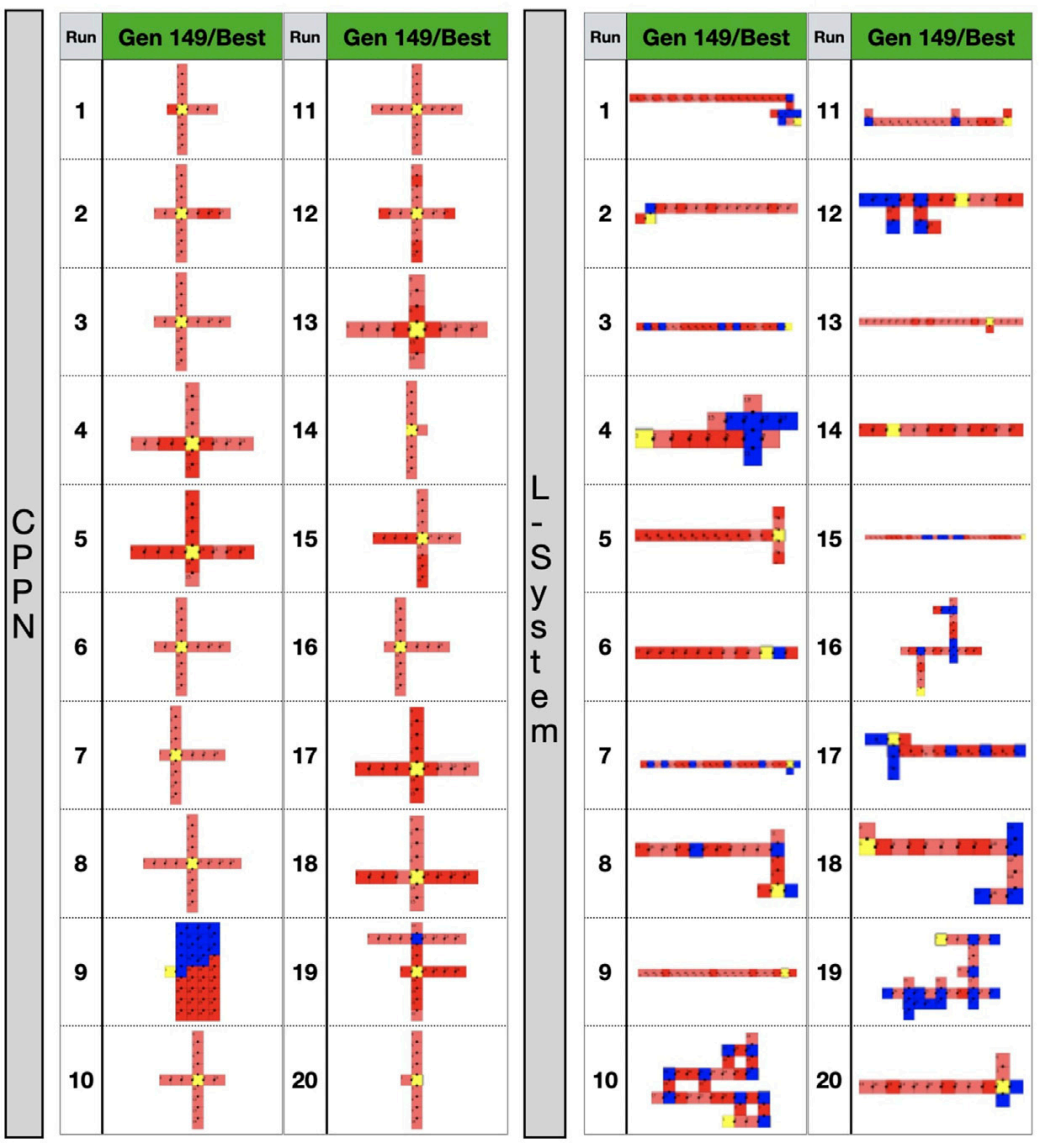
Best robots after optimization in experiment 2.

**FIGURE 13:**
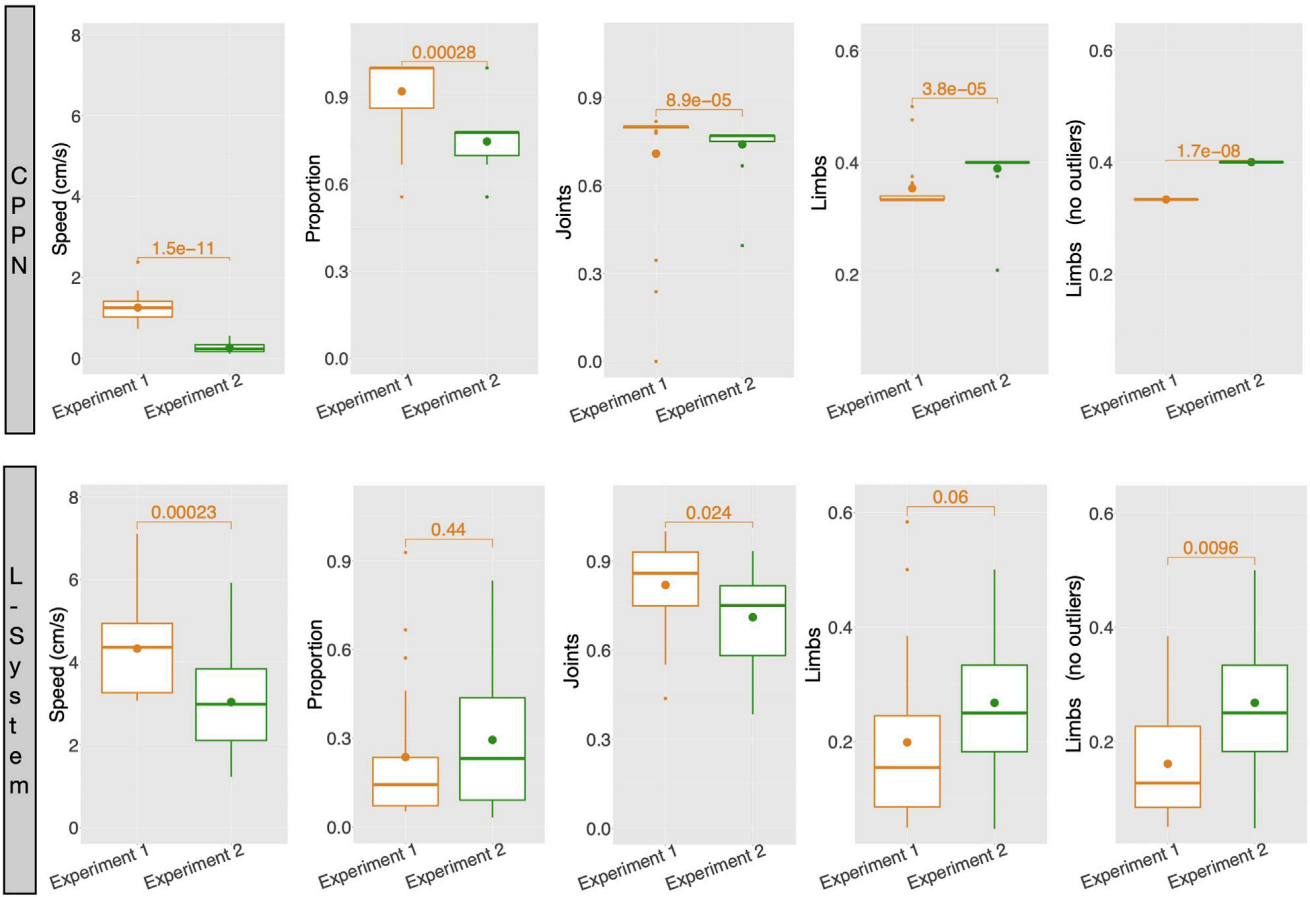
Average robot traits among runs: for each run, includes all robots of the final generation (149) aggregated using the median. Numbers above boxes are *p* values of Wilcoxon tests.

The mechanism of abortion from experiment 2 had more interesting effects on the L-System than on the CPPN. As displayed by Figure 12, complex morphologies emerged with the L-System in some of the runs. Note that by complex, we mean that through visual inspection the shapes appear to be less simple, e.g., multiple limbs to different directions and with different sizes, resulting in walking gaits (as seen in the previous video). Though large snakes are still predominant among the runs, this type of complex morphology was never found with the L-System in experiment 1. With the CPPN, on the other hand, no complex shape emerged in any of the runs. Spiders are still almost always produced, though here they happen more often to be semi-spiders than in experiment 1. Figure 13 provides comparisons between the robots traits of experiment 1—presented earlier—and robot traits of experiment 2 using each encoding. These comparisons support our visual inspection, showing that some robot traits of the populations have changed when using the abortion mechanism. With the L-System, now robots are less actuated and have more limbs—note that the *p*-value for Limbs is slightly above the convention, but becomes quite below it after outliers are removed using the interquartile range. With the CPPN, an increase in limbs and decrease in joints happens as well, with an additional decrease in proportion. This agrees with our observation of the CPPN producing semi-spiders more often than before. As far as performance is concerned, there was a significant drop in speed with both encodings, although this drop was much more dramatic with the CPPN. The knowledge derived from the current experiments does not allow us to formulate clearly a reason for this drop. Still, it seems reasonable to consider that this drop is related to having eliminated the crossover of the L-System and increased mutation probability to the maximum for both encodings. This can be said because preliminary tests have demonstrated that, in terms of performance, the L-System benefits from crossover, while the CPPN benefits from not having crossover and using the mutation probability of experiment 1. The results achieved by this mechanism of abortion are very preliminary, and do not permit drawing strong conclusions. Nevertheless, the purpose of this analysis is to illustrate an alternative to tackling the biases discussed in this paper, considering that the design of an unbiased encoding is still a great challenge.

## 5 Conclusion

In this paper, we experimented with two different generative encodings, namely, CPPN and L-System, and investigated their effects on phenotypic and behavioral robot traits. For robot encodings, we learned that there is a tendency for the two encodings to sample robots with certain traits more often than others, and that the type of robots which are selectively sampled differs between the two encodings. This type of bias derives from the interaction between the encoding with its initialization and reproduction operators. Our results demonstrated that both encodings generate biased samples of robots. More importantly, these biases have a very diverse nature and influence the production of very distinct types of robots. Fundamentally, this observation invites us to reflect on the trade-off that is imposed by such differentiation in the constrained space of each encoding. Robots produced by the CPPN often have a “spider” shape and are relatively slow, but present very coordinated and stable gaits. By contrast, robots produced by the L-System often have a “snake” shape and are much faster, but present exceedingly uncoordinated and unstable gaits. These trade-offs illustrate that producing better robots does not necessarily mean producing robots with higher performance, i.e., faster robots. That is true because “better” could be related to other qualities beyond the performance itself. While one could argue that every desirable trait could and should be reflected in the fitness-function(s), in practice fitness-function design is extremely challenging, specially if too many dimensions need to be optimized. Reflecting on these results, as an alternative to fitness-function design, one could take advantage of such encoding constraints, as a way of driving the search to a particularly convenient solution space. While in some cases a bias could be perceived as a curse that needs to be escaped, in other cases it could also be seen as desirable. That may be the case if, for instance, robots produced with an encoding were biased to a particular trait, and this trait was suitable for their intended environment or task beyond performance. One example: let us imagine robots responsible for carrying water as fast as possible. These robots would need more than speed, but also a balanced and stable gait, so that the water would not be spilt. While designing fitness functions and optimizing for multiple objectives can be tricky, one alternative solution is using an encoding that is very constrained in terms of gait balance, e.g., the CPPN used in the current study. In this case, before making a final choice of encoding and operators, one could experiment with different types, and assess which of them better allow to achieve such convenient solution space.

At this point, it is essential to clarify that the differences we have observed between robots produced by each encoding do not depend simply on the nature of CPPNs and L-Systems as genotypes representations. As mentioned in the Section 3.1, for each one of them we designed our own decoding and reproduction and initialization operators, fitting our design space, and this naturally plays a substantial role in shaping the search space. Therefore, the differences we hereby observed depend on the search space as a whole, including genotype, decoding, initialization operator, and variation operator. That being said, our main message is not about specific biases of such particular combinations, but to demonstrate how these combinations, whatever they are, can constrain evolved robot traits in different ways.

To finalize this discussion, let us recollect that the populations of robots went through an extra stage of initialization aiming to tackle initialization biases. Notwithstanding, though this stage succeeded in increasing diversity in the initial population, the bias persisted through the reproduction operators. This was the case because both of these encodings present problems with locality. Because it is known that generative encodings often suffer from low locality (Rothlauf and Oetzel, 2006), we could hypothesize that the biases originated from low locality are due to both tested encodings being generative. However, related work using this identical robot design space, but with a direct encoding, has verified that a similar bias exists when searching for novelty (Miras et al., 2018b). Whereas in a first moment an obvious solution to that would be improving the encodings so to increase their locality, this is frequently not a trivial endeavor. As one alternative to that, we experimented with evading the biases through an abortion mechanism. This mechanism resulted in interesting effects with the L-System, so that more often complex shapes emerged. The same result was not achieved with the CPPN though, perhaps because its bias is stronger, as we have discussed earlier.

For future work we propose to a) investigate the abortion mechanism in more depth, allowing it to be carried also when there is crossover; b) investigate ways of increasing the locality of both the studied encodings.

## Data Availability

The datasets presented in this study can be found in online repositories. The names of the repository/repositories and accession number(s) can be found in the article/Supplementary Material.
