# Supplementary material for: Constrained by Design: Influence of Genetic Encodings on Evolved Traits of Robots
Source: Front Robot AI. 2021 Jun 15;8:672379. doi: 10.3389/frobt.2021.672379 (PMC8239187; doi:10.3389/frobt.2021.672379)
Supplement: Supplementary file 5 [file Image1.pdf]

Supplementary Material

1 SUPPLEMENTARY DATA

The data of all experiments that we used for the analysis is available on Data S1.

2 VIDEOS

A supplementary video is provided as Video S2, displaying examples of robots in simulation.
